# Europium(ii/iii) coordination chemistry toward applications

**DOI:** 10.1039/d4cc03080j

**Published:** 2024-09-04

**Authors:** Elizabeth C. Lewandowski, Colin B. Arban, Morgan P. Deal, Andrea L. Batchev, Matthew J. Allen

**Affiliations:** a Department of Chemistry, Wayne State University 5101 Cass Avenue Detroit Michigan 48202 USA mallen@chem.wayne.edu

## Abstract

Europium is an f-block metal with two easily accessible oxidation states (+2 and +3) that have vastly different magnetic and optical properties from each other. These properties are tunable using coordination chemistry and are useful in a variety of applications, including magnetic resonance imaging, luminescence, and catalysis. This review describes important aspects of coordination chemistry of Eu from the Allen Research Group and others, how ligand design has tuned the properties of Eu ions, and how those properties are relevant to specific applications. The review begins with an introduction to the coordination chemistry of divalent and trivalent Eu followed by examples of how the coordination chemistry of Eu has made contributions to magnetic resonance imaging, luminescence, catalysis, and separations. The article concludes with a brief outlook on future opportunities in the field.

## Abstract 2

This article describes applications of Eu, including MRI, luminescence, catalysis, and separation science.

## Graphical abstract

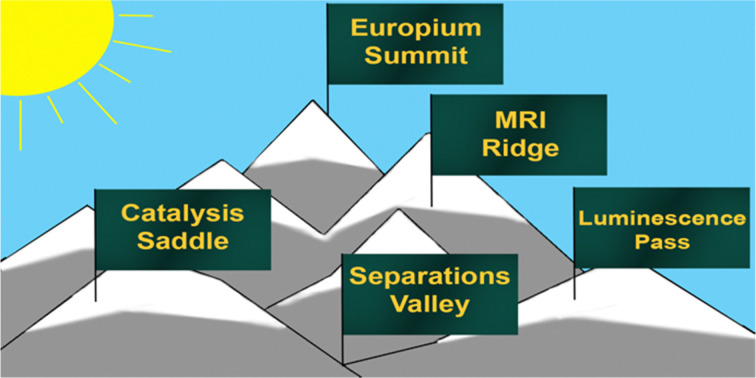

## Introduction

Europium is most stable in the +3 oxidation state but has the most accessible +2 oxidation state of all the lanthanides. Eu prefers to bind to donors like O or N and usually has a coordination number between eight and ten. The valence 4f-orbitals of Eu are shielded from the environment by fully occupied 5s and 5p orbitals, preventing participation of the 4f orbital in bonding. Eu^III^ ions typically give rise to 4f–4f transitions but 4f–5d transitions can occur within the far UV region and are minimally impacted by choice of ligand. However, the 4f-to-5d transitions typically exhibited by Eu^II^ are heavily influenced by ligands. The dramatic differences in electron configurations between ground and excited states of the Eu ions can be thought of as a switch to turn on or off useful magnetic and electronic properties that arise from interactions with the environment. This review describes contributions of the Allen Research Group, and others, regarding the coordination chemistry of Eu^II^ and Eu^III^, how ligands alter the electronic and magnetic properties of the two ions, and the ability to controllably switch between the two ions. These points of discussion are contextualized within the framework of applications in magnetic resonance imaging (MRI), luminescence, catalysis, and the isolation of Eu from other elements.

### Eu^II^ and Eu^III^ coordination chemistry

**Fig. 1:**
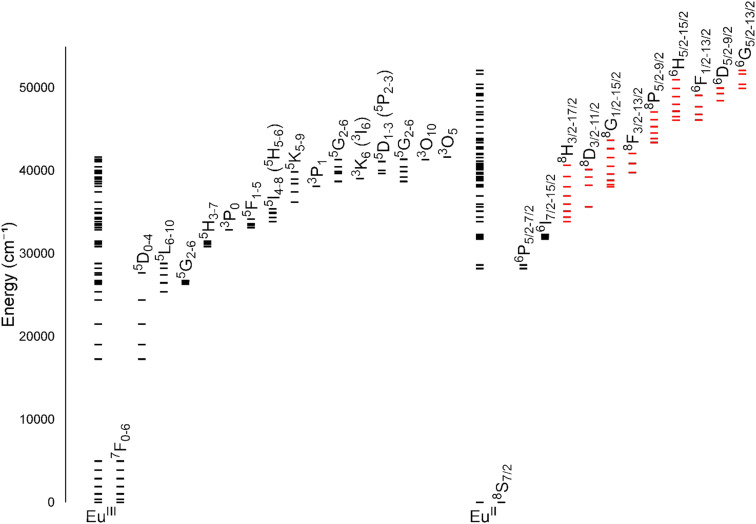
Energy levels for Eu^III^ and Eu^II^. All levels for each ion are shown directly over the ion label. For clarity, subsets of levels are projected to the right of the full diagram and labeled with term symbols. Projected subsets with black lines represent 4f states, and red lines represent 4f5d states. Values for the levels were obtained from previous reports.^1,2^

One of the coarsest ways to control properties of europium is through the oxidation state (+2 *versus* +3). Despite only one-electron difference between Eu^II^ and Eu^III^, their electronic energy states are drastically different from each other (Fig. 1).^1,2^ Eu^III^ has an electron configuration of 4f^6^ with ground-state term symbols of ^7^F_0–6_ and excited states that tend to nonradiatively decay to a long-lived ^5^D_0_ state. Most transitions of Eu^III^ occur between these two energy states,^3^ but these f–f transitions are Laporte forbidden and, consequently, tend to be weak. To increase emission from these transitions, energy transfers to Eu from light-absorbing compounds are used. This process is called the photoinduced electron transfer effect.^4^ Eu^II^ has an electron configuration of 4f^7^ with a ground-state term symbol of ^8^S_7/2_.^5^ Interestingly, Eu^II^ has two common excited state possibilities that result from 4f–4f or 4f–5d transitions. Because the 4f–5d transitions are lower in energy than the 4f–4f transitions, they are more common. Additionally, because 4f–5d transitions are Laporte allowed, they result in intense emissions without the need for photoinduced electron transfer. The dramatic differences in electronic states between Eu^II^ and Eu^III^ influence how the ions interact with ligands and, consequently, the electronic and magnetic properties of Eu-containing complexes.

With respect to divalent europium, the ion largely adopts three coordination numbers: eight, nine, and ten, with eight being the most common. Trivalent europium typically adopts coordination numbers of eight and nine because of the smaller atomic radius of Eu^III^, 1.09 Å, compared to divalent europium, 1.31 Å.^6^ With lanthanides, coordination number and ligand bulk tend to drive geometry. Readers are referred elsewhere to learn about the possible geometry of Eu ions with coordination numbers 8–10.^7–9^ Eu^II^ complexes adopt geometries like bicapped trigonal antiprisms, hula hoops, and tetracapped trigonal prisms.^10–18^ Within the hula hoop and tetracapped trigonal prism geometries, there is often a combination of a multidentate ligand and one or more monodentate coordinated solvent molecules. Overall, the shape of the europium complex depends on the ligand and the space available on the metal coordination sphere.

Beyond coordination number and geometry, hard–soft acid–base properties of ligands are another way to tune the properties of europium. Hard–soft acid–base theory is a way to predict and explain the general types of atoms that bond together. In essence, classifying an atom as either hard or soft is dependent on its polarizability, or its response to an electric field.^19^ Trivalent europium, and other trivalent lanthanides, tend to be thought of as hard acids, but divalent europium is a much softer ion. Consequently, the types of donor atoms used for the two ions tend to be harder donors like oxygen, used more for the trivalent ion, and softer donors like nitrogen and sulfur, used for the divalent ion. The hard–soft properties of donor atoms affect the stability of Eu-containing complexes. For example, sulfur donor atoms shift the oxidation potentials of Eu^II^-containing complexes more positive.^20^ The ability to tune the electrochemical properties of Eu-containing complexes is an important aspect of ligand design for applications in MRI and separation science. However, substantial overlap exists in the identities of ligand donor atoms that will bind to divalent and trivalent ions.

Solvent selection is another important factor to consider when studying Eu-containing complexes. As previously mentioned, solvent molecules coordinate with the Eu ion, influencing electrochemical potentials. For example, the formal potential of Eu^II/III^ is −0.26 V *versus* the calomel electrode in acetonitrile, a weakly coordinating solvent, but the formal potential of Eu^II/III^ is −0.95 V *versus* the calomel electrode in strongly coordinating solvents like hexamethylphosphoramide.^21^ Therefore, when performing studies in solution, selection of solvent is an important consideration.

Finally, when working with complexes of Eu^II^, it is critical to prevent inadvertent oxidation to Eu^III^ by atmospheric O_2_. This prevention involves standard inert atmosphere methods including glovebox and Schlenk techniques and the use of degassed solvents. For applications relevant to MRI, wet gloveboxes are often used for sample preparation in which water is allowed but not molecular oxygen. For many other applications, dry gloveboxes are often used. When preparing samples for solution-phase characterization, including MRI, NMR spectroscopy, and fluorescence or UV-visible spectroscopy, samples need to be sealed to prevent oxygen from entering the sample. Depending on the longevity of the study the samples can be sealed with tape or wax or placed in flame-sealed tubes. For a thorough review of methods to handle divalent europium, readers are referred elsewhere.^22^

## Eu^II^-based contrast agents for MRI

The different electronic and magnetic properties of Eu^II^ and Eu^III^ show potential for responsive contrast agent design in MRI. MRI is an imaging modality that noninvasively generates images based on the relaxation times and chemical shifts of nuclei, most often ^1^H, in a magnetic field. These times and shifts are influenced by paramagnetic molecules called contrast agents, that alter the contrast of images. Clinically, contrast agents are largely used to provide anatomical information, but there is an abundance of research focused on developing contrast agents that provide functional information.^23–28^ Contrast agents for MRI are often metal-containing complexes that consist of a ligand and a paramagnetic ion. The ligand chelates the ion to prevent dissociation but enables interaction with the environment, for example with water molecules. Current clinically approved contrast agents include a paramagnetic ion, like Gd^III^, to enhance image contrast by decreasing ^1^H nuclear relaxation times. The ability to enhance contrast is measured in terms of relaxivity that has units of mM^−1^ s^−1^. Properties like electronic relaxation time, magnetic moment, and unpaired electrons are well suited for relaxing ^1^H nuclear spins, which are important for consideration in the design of contrast agents for MRI.^29,30^ Aside from Gd^III^-containing complexes, contrast agents using other paramagnetic metals such as Mn^II^, Fe^III^, and Eu^II^ have also been explored.^31–33^ Interest in Eu^II^-based contrast agents has arisen because Eu^II^ and Gd^III^ are isoelectronic and have similar magnetic properties that are useful in MRI.^34^ The use of Eu is well suited for inclusion in responsive imaging probes because of the orthogonal imaging properties of Eu^II^ and Eu^III^, enabling complexes of the ions to function as imaging reporters of oxygen concentration.

**Fig. 2:**
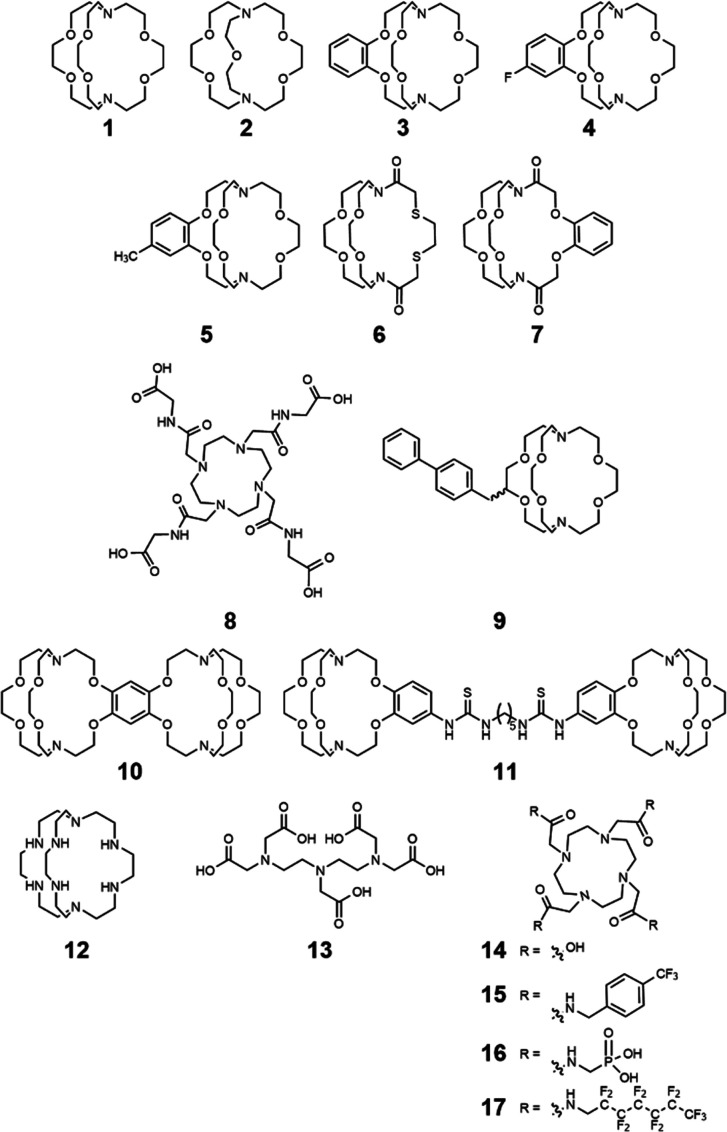
Selected ligands relevant to Eu-based contrast agents for MRI.

Studying aqueous coordination chemistry is critically important for applications involving aqueous solutions of europium-containing complexes. An important factor of a contrast agent is that it needs to be water soluble and coordinate with rapidly exchanging water molecules. For divalent europium, short metal–water distances, multiple coordinated water molecules, and optimal water exchange rates increase contrast enhancement. However, slow dissociation rates are also important to avoid europium dissociation from complexes. Ultimately, a balance of properties is required to tune all of these interconnected aspects of coordination chemistry. Ligands described in this section that influence coordination chemistry relevant to MRI are depicted in Fig. 2. This focus is shared by the Allen Research Group in the study of europium complexes.

### Ligand characteristics and electrochemical potentials

When thinking about ligands to bind Eu^II^ in potential contrast agents, the size of the ligand binding pocket has a critical role. This concept was explored by observing differences between a 2.2.2-cryptand, 1, and a 2.2.1-cryptand, 2, with differences in the size of the cryptand cavity.^35,36^ Ligand 2 is smaller than 1 by the difference of two carbon atoms and an oxygen atom, reducing the cavity size. The stability of Eu-containing complexes of 1 and 2 differs depending on the oxidation state of the Eu ion. The dissociation of Eu^II^-containing cryptates are faster than those of the corresponding Eu^III^-containing cryptates.^35,36^ These trends related to cavity size and oxidation state are translatable to other ligands. Overall, study of the kinetic rates and thermodynamic stability of Eu-containing complexes is relevant to MRI because ligands need to possess the ability to coordinate Eu both before and after oxidation from Eu^II^ to Eu^III^.

One useful technique for determining dissociation constants is UV-visible spectroscopy; however, the absorption spectra of Eu^II^-containing complexes and the Eu^II^ aqua ions often overlap, limiting the ability to monitor dissociation. Inspired by UV-visible studies of murexide with other metals,^37,38^ murexide was studied as an indicator to aid in the measurement of dissociation rates of Eu. Using Eu^II^1 and murexide, absorption measurements at 485 nm were recorded as a function of time, and the absorption values were used to determine the concentration of Eu^II^.^15^ The natural log of the concentration *versus* time indicated a first order relationship with the slope of the plot revealing the dissociation constant. Results from the absorption spectra analysis were consistent with the results determined from electrochemical techniques. When comparing Eu^II^1 and Eu^II^3, dissociation rates increased upon addition of a benzo group to the complex. Differentiation between functional group additions to benzo group complexes is seen when comparing Eu^II^4 and Eu^II^5. The results show that the addition of a variety of electron-withdrawing groups can be differentiated when using murexide with UV-visible spectroscopy to determine dissociation rates. Overall, this study demonstrated that murexide can be used to measure dissociation rates of Eu^II^-complexes. The murexide indicator adds to the toolbox of methods for determining dissociation rates of Eu^II^-containing complexes. Additionally, the inertness of Eu^II^-containing cryptands was studied with respect to transmetallation by endogenous ions such as Ca^II^, Mg^II^, and Zn^II^ based on previous studies.^39^ Along these lines, Eu^II^1, Eu^II^3, Eu^II^6, and Eu^II^7 were studied in the presence of Ca^II^, Mg^II^, and Zn^II^ and found that Eu^II^-containing cryptates without amide groups are kinetically stable in the presence of the ions.^40^

Another important aspect of Eu chemistry relevant to contrast agents is the electrochemical potential, which needs to be more positive than the electrochemical potential of water to avoid reduction of water by Eu^II^. Eu^II^-containing complexes in aqueous solutions were studied to report the complexes that are most oxidatively stable.^20^ Results demonstrated that ligand modifications to the hard–soft acid–base properties of donor atoms shifted the oxidative potential of Eu^II^. Additionally, the electrochemical behavior of Eu-cyclen-based complexes was studied with various ratios of glycinamide to acetate arms.^41^ It was found that the substitution of an amide for a carboxylate donor in the coordination environment led to more positive oxidation potentials of Eu^II^. The results from this investigation showed the significance of the ligand design in the tuning of the electrochemical potentials of the Eu-containing complexes that are used for redox-responsive contrast agents.

Understanding the thermodynamics and kinetics of oxidation is important to aid in the rational design of new complexes for the potential use of oxidatively responsive Eu^II^-containing complexes *in vivo*. Toward this goal, the oxidation of Eu^II^-containing complexes, Eu^II^4, Eu^II^8 and EuCl_2_ was studied.^42^ The oxidation of Eu^II^ by molecular oxygen was observed in varying pH conditions because more acidic media results in slowed oxidation rate.^34^ Interestingly, the largest oxidant studied, glutathione disulfide, was unable to oxidize Eu^II^, hinting that it is possible to kinetically control oxidation. This kinetic control would later be demonstrated by the Allen Research Group with ligand selection instead of oxidant selection.^43^

### Relaxivity

A key measure of whether a complex will serve as an effective contrast agent is relaxivity. Large relaxivities are desirable because they enable small limits of detection. Variations in structural characteristics of ligands can tune Eu^II^ properties that influence relaxivity. As previously mentioned, some of these properties include water-exchange rate, water-coordination number, and molecular reorientation time.

Other groups have demonstrated that the Eu^II^ aqua ion and cryptate have outstanding water-exchange properties for potential use in contrast agents for MRI.^34,44,45^ One report illustrates the relationship between relaxivity and the molecular weight of Eu^II^-containing cryptates, Eu^II^1, Eu^II^3, and Eu^II^9.^46^ The relaxivity of Eu^II^1, Eu^II^3, and Eu^II^9 increases as a function of molecular weight at all field strengths, consistent with complexes of Gd^III^.^47^ Further, the correlation between slowing molecular tumbling rates and relaxivity through covalent and noncovalent interactions with macromolecules were investigated.^15,48^ In this study, cryptate Eu^II^9 was expected to form noncovalent inclusion complexes with cyclodextrins and albumin to increase relaxivity by slowing the molecular tumbling rate. It was found that the relaxivity of Eu^II^9 in the presence of the macromolecules increased relaxivity as a function of the molecular weight of the macromolecules; however, the increase in relaxivity was smaller than expected based purely on molecular weight due to internal rotations.

To address the disparity between molecular weight and rotation, a rigid linker was incorporated to bridge two ions of Eu^II^ to study the relationship between rotational dynamics and relaxivity.^18^ Comparison of the rotational dynamics with the relaxivity of the contrast agents was done by comparing Eu^II^10, a rigid complex, with Eu^II^11, a Eu^II^-containing complex that can rotate freely around the single bonds of the linker between two Eu^II^ ions. The nuclear magnetic relaxation dispersion data shows local rotation of Eu^II^11 stemming from the rotation of about the flexible linker between the two cryptates. The more rigid linker in Eu^II^10 correlated to longer global rotational correlation time, resulting in an increase in relaxivity. These results demonstrate that rigidity in linking Eu^II^ to other molecules is beneficial for increasing the relaxivity of redox-active, Eu^II^-based contrast agents.

**Fig. 3:**
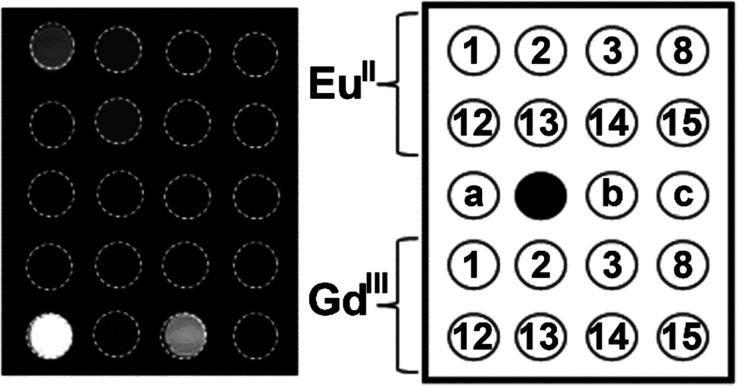
*T*_1_-weighted MR image of a multiwell plate of solutions of Eu^II^1–3, Eu^II^8, Eu^II^12–15 and Gd^III^1–3, Gd^III^8, Gd^III^12–15 at ambient temperature. Label a represents phosphate-buffered saline, b denotes GdCl_3_, and c represents EuCl_2_. Adapted from Corbin *et al.*,^49^ copyright 2018, with permission from Elsevier.

The knowledge of Eu^II^ relaxivity and oxidation kinetics was integrated with coordination chemistry to enable screening of new ligands.^49^ The rational design of ligands to complex both oxidation states of Eu is important to prevent dissociation upon oxidation of the metal; this design requires a method to detect ligands that can bind both the soft, large Eu^II^ ion, as well as the harder and smaller Eu^III^ ion. The method involves complexation of ligands to both Eu^II^ and Gd^III^, in which Gd^III^ is used as an MRI-active surrogate for Eu^III^. In the method, metals are mixed with ligands, and uncomplexed metal is precipitated with phosphate buffer. The resulting mixtures are filtered into a multiwell plate that is imaged using MRI, with bright spots in the image indicating that a contrast-enhancing complex was formed. Additionally, for Eu^II^-containing complexes, a bright spot indicates that a complex was formed that does not reduce water. Dark spots in the images indicate lack of coordination with the ligand or the formation of a Eu^II^-containing complex that reduced water to form Eu^III^. To test the screening procedure, a set of eight ligands, 1–3, 8, 12–15, were selected due to the range of ability of the ligands to coordinate Eu^II^ and Gd^III^. The screening procedure was able to provide binding information (Fig. 3),^49^ and was subsequentially used in future studies.^43^

### *In vivo* studies

**Fig. 4:**
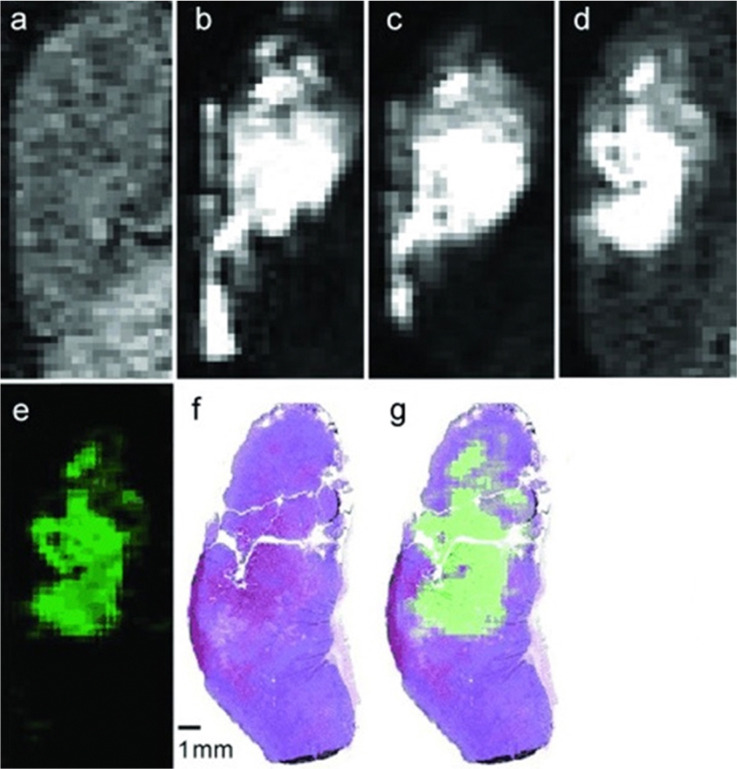
*T*_1_-weighted *in vivo* sagittal plane images of a 4T1 tumor injected with Eu^II^4. Image (a) is before the injection and (b) is 3 min, (c) is 20 min, and (d) is 120 min post-intratumoral injection. Image (e) is the difference between images (d) and (a). Image (f) is a hematoxylin- and eosin-stained slice of the tumor corresponding to images a–e. Figure (g) is an overlaid image of images (e) and (f). Used with permission of John Wiley & Sons, from A Eu^II^-Containing Cryptate as a Redox Sensor in Magnetic Resonance Imaging of Living Tissue, L. A. Ekanger, L. A. Polin, Y. Shen, M. E. Haacke, P. D. Martin, M. J. Allen, Volume 54, Copyright 2015; permission conveyed through Copyright Clearance Center, Inc.

Although Eu^II^ was proposed as a redox-active contrast agent around the turn of the century,^34^ no studies of its use *in vivo* had been reported before 2015.^13^ Based upon *in vitro* findings, *in vivo* imaging using Eu^II^-containing complexes was pursued. The first report of a Eu^II^-based complex demonstrating an ability to serve as an oxygen-responsive contrast agent *in vivo* was reported using tumor-bearing and healthy mice.^13,50^ These initial reports involved two studies: one studying direct injection into hypoxic tumors (Fig. 4)^13^ and one looking at different injection sites to understand rates of oxygen diffusion.^50^ Across both studies, areas of less oxygen diffusion corresponded to longer imaging times (up to several hours for hypoxic tumors) and areas of more oxygen diffusion corresponded to nearly instantaneous oxidation (intravenous injections). The ability to differentiate between hypoxic and normoxic tissues has the potential to aid in the study of many diseases. Despite the benefits of having an oxygen-responsive contrast agent based on Eu^II^, these studies are limited by lack of Eu^III^ imaging after Eu^II^ is oxidized and by the need for direct injection into sites of interest.

### Kinetic approaches for slowing Eu^II^ oxidation

One of the limitations preventing systemic delivery of Eu^II^-containing contrast agents is their lack of persistence in oxygenated solutions. This limitation confines the use of Eu^II^ to injection directly into sites of interest, which is not always feasible. For example, the locations of potential hypoxic regions of interest are not always known prior to imaging. To overcome this limitation, persistence of Eu^II^ in oxygenated environments needs to occur. Previous studies of Eu^II^-containing complexes for MRI of hypoxia focused on increasing relaxivity and thermodynamic stability by altering the coordination chemistry of Eu^II^; however, to enable delivery followed by imaging, slowing the oxidation rate of Eu^II^ became a target of research.

The first example of persistence of Eu^II^ in oxygenated solution stemmed from the study of 21 ligands with varying denticities, sizes, donor atom identities, and cavity sizes that were screened for ability to coordinate both Eu^II^ and Eu^III^.^43^ From this screening process, a tetraamide phosphonate-containing ligand, 16, was identified as being able to coordinate to both Eu^II^ and Eu^III^,^43^ and was further investigated. Interestingly, previous studies with 16 demonstrated pH-dependent relaxivity with Gd^III^.^51–54^ The pH-dependency was of interest to study based on other Eu^II^ studies of tetraamine ligands showing temperature-dependent cage formation, contrast enhancement, and slowed oxidation of Eu^II^.^55^ If similar cages are formed with the phosphonate groups to trap coordinated water molecules, those cages could sterically interfere with the approach of oxygen to slow oxidation, similar to how glutathione could not oxidize Eu^II^8 despite being thermodynamically possible.^42^ Studies of Eu^II^16 were performed to understand if the phosphonate groups could slow oxidation of Eu^II^ as a function of pH.^43^ UV-visible measurements were performed from pH 7 to 10 to understand Eu^II^ persistence within Eu^II^16. As pH increased, the persistence of Eu^II^ increased, with Eu^II^ half-lives of 6.4 and 12.4 minutes at pH 7 and 10, respectively. Those pH values surround one of the p*K*_a_ values of phosphonates.^56,57^ Consequently, it was hypothesized that the longer persistence of Eu^II^ in basic solution is due to a cage-like formation of 16, trapping an innersphere molecule of water as a function of the protonation state of the phosphonates. Because of the oxygen resistance observed in the UV-visible measurements, Eu^II^16 was used for systemic delivery studies in healthy mice that were monitored using dynamic contrast-enhanced MRI.^43^ Eu^II^16 was injected into the tail vein of mice, and contrast-enhanced signal was monitored over time using MRI. The half-life of the contrast enhanced signal is roughly seven minutes, which is consistent with *in vitro* studies at neutral pH. Overall, this study introduces a new Eu^II^-containing complex that showed persistence in oxygenated solutions during *in vitro* and *in vivo* measurements, which is an important step toward systemic delivery of Eu^II^ contrast agents for detection of hypoxia.

**Fig. 5:**
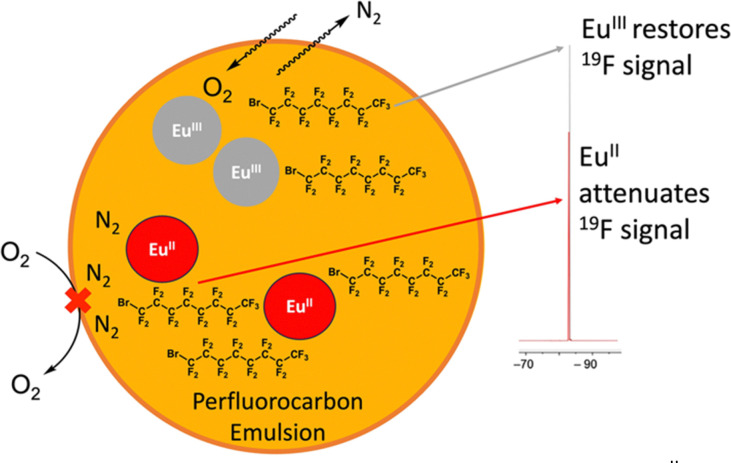
Cartoon representation of Eu^II^17 in lecithin/perfluorocarbon emulsion. The orange represents a perfluorocarbon mixture surrounding Eu^III^17, the grey circle, and Eu^II^17, the red circle.

In addition to increasing persistence using coordination chemistry of phosphonate-containing ligands, an outersphere approach toward increasing persistence was pursued using highly fluorinated ligands in perfluorocarbon nanoemulsions.^58^ Within this study, Eu^II^ was complexed to a perfluorinated, cyclen-based ligand, 17, and subsequently dispersed in an N_2_-saturated perfluorocarbon/lecithin nanoemulsion to slow oxygen diffusion toward Eu^II^ (Fig. 5). The multiple interfaces between water and lecithin and lecithin and N_2_ surrounding perfluorocarbons were hypothesized to slow oxygen diffusion to Eu^II^ based on reports of gas diffusion across interfaces.^59,60^ Additionally, saturation of the perfluorocarbon emulsion with N_2_ was also hypothesized to slow diffusion of oxygen in the vicinity of Eu. The perfluorinated ligand and nanoemulsion are useful for detection of Eu^II^ concentration *via*^19^F-MRI measurements because ^19^F-signal increases as Eu^II^ concentration decreases due to Eu^II^ line broadening effects.^58^ To understand the relationship between ^19^F-signal and Eu^II^, ^19^F-NMR measurements of Eu^II^17 and Eu^III^17 in equimolar concentrations were acquired in degassed and oxygenated perfluorocarbon mixtures. These studies revealed that the oxidation state of Eu^II^ is responsible for almost all the signal change in ^19^F-NMR measurements. Contrast enhanced signal of solutions of Eu^II^17 within a perfluorinated nanoemulsion and a solution of Eu^II^15 were measured within thigh muscles of healthy mice using MRI measurements. The perfluorinated nanoemulsions of Eu^II^17 revealed persistence of Eu^II^ in detectable amounts for at least 30 minutes (compared to <5 minutes for a control complex), indicating that the nanoemulsion is able to slow the diffusion of oxygen to Eu^II^. This study represents an important step toward increasing the persistence of Eu^II^ signal through an outersphere approach. Overall, these studies show how the surrounding environment of Eu^II^ can influence Eu^II^ persistence in oxygenated solutions, which is an important step toward hypoxia detection using MRI *via* systemic delivery of contrast agents.

### Multimodality

With the ability of Eu^II^-based contrast agents to generate images *in vivo*, limitations arise from the lack of the ability to image the Eu^III^ ion after oxidation of Eu^II^. As mentioned previously, contrast enhancement of Eu is not observed in the oxidized Eu^III^ state, so methodology for designing a contrast agent that can functionally image the trivalent oxidation state is important. The strategy that the Allen Research Group has taken is to study modalities that have demonstrated the ability to image Eu^III^ with other systems and then try to combine those modalities with MRI of Eu^II^. Additionally, the use of multimodal probes can overcome the concentration dependency of responsive contrast agents.^61^ The remainder of this section highlights some of those attempts.

**Fig. 6:**
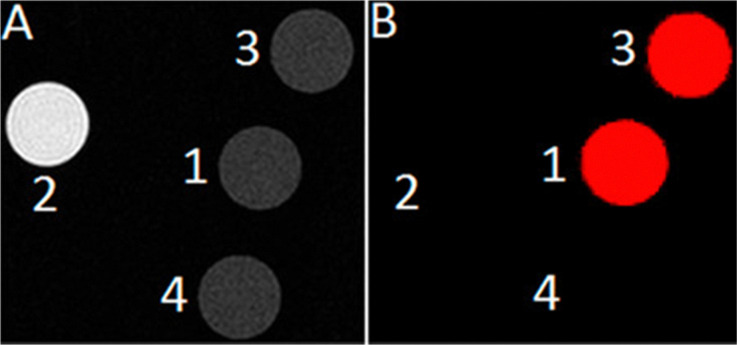
Image (A) is a *T*_1_-weighted image, and image (B) is a CEST difference image. For both (A) and (B), spot 1 represents Eu^III^8, spot 2 represents Eu^II^8, spot 3 represents Eu^III^8 from the oxidation of Eu^II^8, and spot 4 represents water. Reprinted with permission from L. A. Ekanger, D. R. Mills, M. M. Ali, L. A. Polin, Y. Shen, E. M. Haacke and M. J. Allen, *Inorg. Chem.*, 2016, **55**, 9981. Copyright 2016 American Chemical Society.

One study involved encapsulation of a Eu^II^-containing complex within liposomes to create a dual-mode contrast agent that is oxidation-responsive, and upon oxidation of Eu^II^ to Eu^III^ would result in a probe for chemical exchange saturation transfer (CEST) MRI.^62^ CEST is a method of using exchangeable protons to create images using MRI.^63,64^ Liposomes were selected because the inner cavity can hold water-soluble contrast agents, increasing the ratio of water protons associated with the liposome to bulk water protons. The designed liposome system uses *T*_1_ enhancement that visualizes the presence of the Eu^II^ oxidation state along with CEST that visualizes both Eu^II^ and Eu^III^.^61^ From observation of the potential outcomes from the CEST and *T*_1_ measurements, the oxidation state of the Eu ion can be determined without knowledge of the concentration of Eu. The liposome encapsulated Eu^II^-containing complex enabled the observation of the first oxidation-responsive dual-mode contrast agent designed around the oxidation state of Eu. Moving from liposomes to small molecules as CEST probes, the use of Eu^II/III^-cyclen based derivatives as oxygen-sensitive MRI contrast agents was explored.^65^ In this study, Eu^II^8 provides *T*_1_-weighted contrast enhancement but no CEST signal, and Eu^III^8 produces CEST signal but no *T*_1_-weighted signal enhancement (Fig. 6). The small molecule probes were characterized by a variety of methods to characterize both oxidation states of the complex. Ultimately, the ability to image before and after oxidation of Eu^II^ was demonstrated.

Another system studied as potential multimodal contrast agent integrated MRI and photoacoustic imaging techniques.^66^ In this system, MRI has the ability to scan large areas and photoacoustic imaging has the ability to create images of areas that are accessible by light. To determine the potential of having a redox-responsive multimodal contrast agent for both MRI and photoacoustic imaging, Eu8 was selected based on previous studies demonstrating that the divalent complex is yellow in color, to enable absorption for photoacoustic imaging, and that it is an effective contrast agent for MRI.^65^ Eu8 was effectively able to function as a multimodal contrast agent; however, because both modalities could only be detected with Eu^II^ and not Eu^III^, the system was not ideal for overcoming the challenge of detecting Eu^III^.

In addition to CEST MRI, the use of ^19^F signals can be combined with ^1^H MRI in multimodal probes, because ^19^F MRI has been used *in vivo* in various applications.^67–70^ A major concern of using ^19^F signal is finding the amount of fluorine that enables solubility of Eu^II^-containing complexes in water and results in detectable signal. One study aimed to address this concern by studying changes based on the number and placement of fluorine atoms in a set of ligands.^55,71^ One of the ligands, 15, contains four *p*-trifluoromethylbenzyl groups that when complexed with Eu form a pocket where a coordinated water molecule is caged in the solid state.^55^ The trapping of the water molecule is unique compared to other tetraamide complexes that have arms that point away from each other instead of toward each other.^72–74^ Oxidation of Eu^II^ to Eu^III^ enables the presence of an observable ^19^F signal and the detection of the Eu^III^-containing complex.^55^ The ratio of ^19^F and ^1^H signals could also be potentially used for ratiometric imaging beyond just observing Eu^III^ after oxidation of Eu^II^.^71^ Ratiometric imaging is important because of its ability to lead to quantification of medically relevant biomarkers like oxygen concentration in hypoxic environments. Quantification of hypoxia is difficult because the MRI signal produced using Eu^II^-based contrast agents is dependent on the concentrations of oxygen and Eu^II^. Based on previous studies involving multimodal imaging and the relationship of ^19^F signal and Eu^II^ signal, levels of hypoxia can be determined ratiometrically using a fluorinated Eu^II^-based contrast agent.^71^ and comparing the ^1^H and ^19^F-MRI signals produced from a dual-mode redox responsive contrast agent.^64^ In this study, hypoxia was detected by plotting the *T*_1_-weighted ^1^H-MRI signal with ^19^F-MRI signal. These results enable quantification without the knowledge of the concentration of Eu.

## Eu in luminescence

**Fig. 7:**
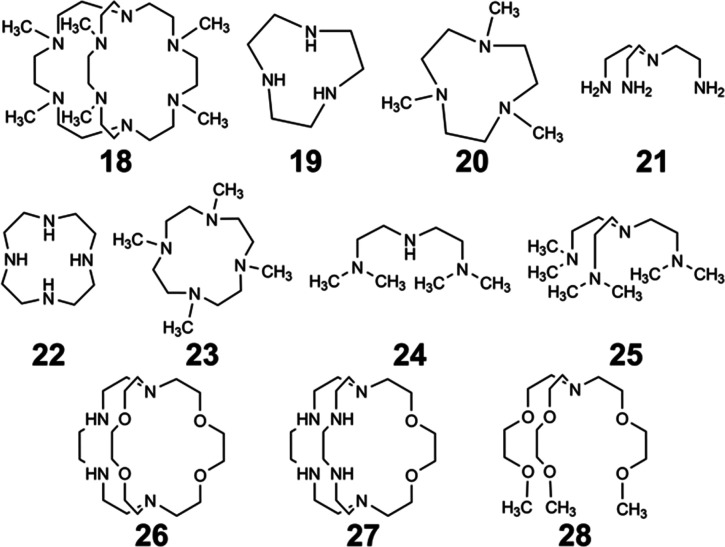
Ligands used in luminescence-based studies.

Within the Allen Research Group, interest in Eu^II^ luminescence stemmed from the discovery of Eu^II^-aza-[2.2.2]-cryptate,^75^ Eu^II^12, that occurred during the search for new contrast agents for MRI. Luminescence refers to the emission of light after a molecule has been excited by a specific source like heat, light, or energy transfer. The luminescence of Eu ions is relevant to lighting, electronic screens, and imaging. Further, the luminescence of both Eu^II^ and Eu^III^ can be tuned with ligand design and coordination chemistry. Within this section, general information about the orbital transitions relevant to Eu^II^ luminescence involving ligand design studies for Eu^II^ and Eu^III^ are described. Ligands described in this section that influence coordination chemistry relevant to luminescence are depicted in Fig. 7. For detailed descriptions of the luminescence of Eu^II^ and Eu^III^, readers are referred elsewhere.^3,76–78^

### 4d–5f transitions of Eu^II^-containing complexes

The broad electromagnetic spectra of Eu^II^-containing complexes in the UV-visible range arise from 4f^6^–5d^1^ transitions that are tunable using coordination chemistry. Before understanding how ligands affect these transitions, it is helpful to describe some general information about 4f^7^–4f^7^ and 4f^6^–5d^1^ transitions of Eu^II^. Eu^II^ undergoes 4f^7^–4f^7^ transitions, manifested as narrow, low-intensity peaks in luminescence spectra and 4f^6^–5d^1^ transitions that give rise to broad, intense peaks. Often because of the difference in peak intensities, the 4f^6^–5d^1^ transitions obscure 4f^7^–4f^7^ transitions.^78^ Although 4f^6^–5d^1^ transitions are broad, the intensity of these peaks can be quenched by nonradiative decay when OH and NH oscillators are within the Eu^II^ coordination sphere. Because OH and NH oscillators decrease the intensity of Eu^II^ luminescence, it is useful to avoid solvents and ligands containing OH and NH groups. Overall, the diffuse nature of the 5d orbitals relative to the 4f orbitals, which are shielded from the environment, enable tuning of the 4f^6^–5d^1^ transitions using coordination chemistry. For more information describing the Eu^II^ orbital transitions and energy splitting, readers are referred elsewhere.^5,79–82^

### Divalent Eu complexes used in luminescence studies

**Fig. 8:**
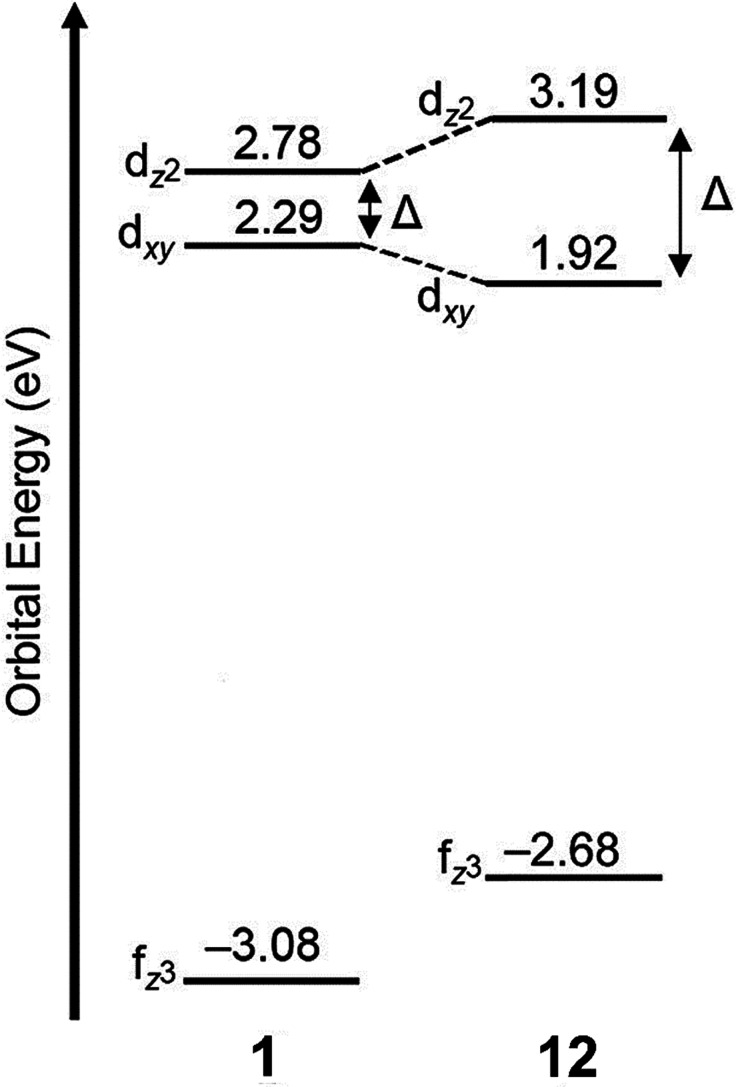
Calculated d-orbital splitting of Eu^II^1 and Eu^II^12. Adapted from Corbin *et al.*,^81^ copyright 2018, with permission from Elsevier.

As mentioned earlier, Eu^II^12, was initially pursued as a potential contrast agent for MRI. Ligand 12 was synthesized to be structurally similar to [2.2.2]-cryptand, 1, but with N donors instead of O donors. Using N donors instead of O donors was targeted to tune the hard–soft acid–base matching between Eu^II^ and the ligand to tune the Eu^II/III^ redox couple. Crystal structures of Eu^II^1 and Eu^II^12 both reveal nine-coordinate geometries in which all donor atoms and one counterion coordinate to Eu^II^.^14,75^ However, despite the similarities in the solid state, Eu^II^12 shows extremely different emission wavelengths and quantum yields in solution compared to Eu^II^1. The excitation and emission maxima of Eu^II^12 are 415 and 580 nm, respectively, and Eu^II^1 has excitation and emission maxima of 259 and 471 nm, respectively.^81^ The emission wavelength of Eu^II^12 is shifted relative to Eu^II^1, likely due to lower energy 4f–5d transitions. The quantum yield of Eu^II^12 is 26% in basic aqueous solution and 37% in methanol.^82^ The crystal structure of Eu^II^12 reveals a chloride ion in the inner coordination sphere that is in a position to form hydrogen bonds with NH groups of the ligand.^75^ Conductivity studies to understand the coordination of Cl^−^ to Eu^II^12 in solution showed that Cl^−^ is likely coordinated to Eu^II^ in aqueous solution. The chloride ion enables high quantum yield because it blocks coordination of nonradiatively quenching OH oscillators in the innersphere of Eu^II^12 in basic aqueous solution. The aqueous coordination of Cl^−^ to Eu^II^12 is explained using hard–soft acid–base theory because Eu^II^ is a soft ion and coordinates better to Cl^−^ than OH^−^.^19,83^ The differences in luminescence between the two complexes is explained through computational studies comparing the differences of Eu^II^1 and Eu^II^12.^81^ The computational calculations indicate that Eu^II^1 and Eu^II^12 have similar excitation spectra but different emission spectra because Eu^II^12 undergoes a geometry change to a more stable conformation. The geometry change in Eu^II^12 increases the splitting of Eu^II^ d-orbitals and results in a more red-shifted emission spectra by bringing the 4f and 4d orbital energies closer together than without the geometry change in Eu^II^1 (Fig. 8). The initial report of Eu^II^12 opened several studies in the Allen Research Group focused on exploring the luminescence of Eu^II^12 and other selected complexes.

Because of the seemingly important role of the chloride ligand in the luminescence of Eu^II^12, one subsequent study of Eu^II^12 investigated how the identity of counterions influences excitation and emission peaks.^10^ Ligand 12 was used to occupy eight coordination sites of Eu^II^ so only one coordination site was left for the following ions: Cl^−^, Br^−^, I^−^, and PF_6_^−^. Initial luminescence measurements were performed in methanol and showed similar absorbance and emission values for each counterion in solution. Acetonitrile was also used because it is a weaker ligand than methanol, decreasing competition between solvent and counterion for binding to Eu^II^.^84^ In acetonitrile, a blue shift in emission spectra is observed with Cl^−^ and Br^−^ counterions in Eu^II^12 relative to the same complexes in methanol, whereas I^−^ and PF_6_^−^ counterions do not result in changes in emission from methanolic solutions.^10^ The blue shift arising from Cl^−^ and Br^−^ is attributed to those ions binding more preferentially to Eu^II^ than acetonitrile. The I^−^ and PF_6_^−^ ions are less likely to coordinate,^84^ in which case acetonitrile coordinates to Eu^II^12 to complete a nine-coordinate geometry or no monodentate ligand coordinates, resulting in an eight-coordinate geometry. This study demonstrates the importance of counterions and solvent selection for Eu^II^ luminescence measurements.

Another avenue of study of the luminescence of Eu^II^ explored the effect of changing the secondary amines in 12 to tertiary amines.^11^ In this study, the secondary amines of 12 were methylated to yield 18 that has increased steric bulk and slightly stronger donors compared to 12. The initial expectation with 18 was that the tertiary amine donors would split the d-orbitals of the Eu^II^ complex more than the secondary amines within 12, resulting in more red-shifted emissions with Eu^II^18 than with Eu^II^12. Additionally, the lack of NH oscillators on the ligand and steric bulk was expected to increase luminescence quantum yield by minimizing pathways for nonradiative quenching. The crystal structure of Eu^II^18 revealed that the methyl groups blocked all inner sphere coordination sites for monodentate ligands, resulting in Eu^II^ adopting an eight-coordinate geometry in the ligand. Upon studying the luminescence of Eu^II^18, emission wavelengths are more shifted toward the UV region compared to the emission wavelengths of Eu^II^12. A computational study suggested that the change in geometry was the dominant factor that regulated the emission wavelength. Despite the unexpected luminescence wavelength, the quantum yield of the Eu^II^18 was 47%. This quantum yield was the largest reported for Eu^II^ in aqueous solution at the time and is due to the methyl groups on the tertiary amines shielding Eu^II^ from luminescence quenchers in solution. This study highlights the importance of geometry and the effect of quenchers on the luminescence of Eu^II^.

In an attempt to understand what components of 12 were needed to control the luminescence of Eu^II^, several ligands of smaller denticity that represent parts of 12 were studied with Eu^II^.^85^ Within this study, absorbance measurements were compared of Eu^II^ in the presence of nineteen ligands of varying degree of functionalization, denticity, and structure. When comparing degree of functionalization of amines between ligands 19–25, the secondary amines on linear and macrocyclic ligands bathochromically shift the absorbance of Eu^II^ more than primary and tertiary amines. The absorbance of Eu^II^ did not shift greatly when comparing linear and macrocyclic ligands 20 and 23–25, except for an additional shoulder reaching into the blue-light region for 23. Finally, increasing denticity of both macrocyclic and linear ligands further blue-shifted absorbance spectra of Eu^II^, likely due to the chelating effect. Overall, secondary amines, macrocyclic ligands, and increasing denticity shifted the emission maxima from the UV toward the blue region. This study compared ligand characteristics necessary to understand and control the luminescence of Eu^II^, and it is an important contribution toward rational ligand design.

Outside of the Allen Research Group, others have investigated how varying donor atoms within ligands affect emission maxima, luminescence lifetime, and redox potential of Eu^II^. Liu, Bian and coworkers, compared 1 against ligands with fewer oxygen donors, resulting in ligands 26 and 27.^86^ The photophysical properties in the solid state and in solution were studied of each Eu^II^ complex. Absorption and emission maxima of Eu^II^-complexes in methanol shifted toward the blue-light region when ligands had fewer oxygen donors and more nitrogen donors. Specifically for emission maxima, there was a shift of 19 nm toward the blue region when the number of N donors was increased, consistent with a greater d-orbital splitting of Eu^II^. Luminescence lifetime of Eu^II^ also increased with more N donors than O donors when in methanol. Luminescence lifetime also increased with more N donors by ∼175 ns because as the number of N donors increase, the excited state of Eu^II^ is stabilized. Conversely, emission maxima and luminescence lifetime of Eu^II^ with ligands containing more O donors decreased. Overall, an increasing, linear trend was found between the number of O and N donors and the emission maxima and luminescence lifetime of Eu^II^ complexes. Within this study, the different effects of N and O donors for Eu^II^ luminescence were compared to understand the tunability of Eu^II^ properties that are important for light-emitting diodes.

**Fig. 9:**
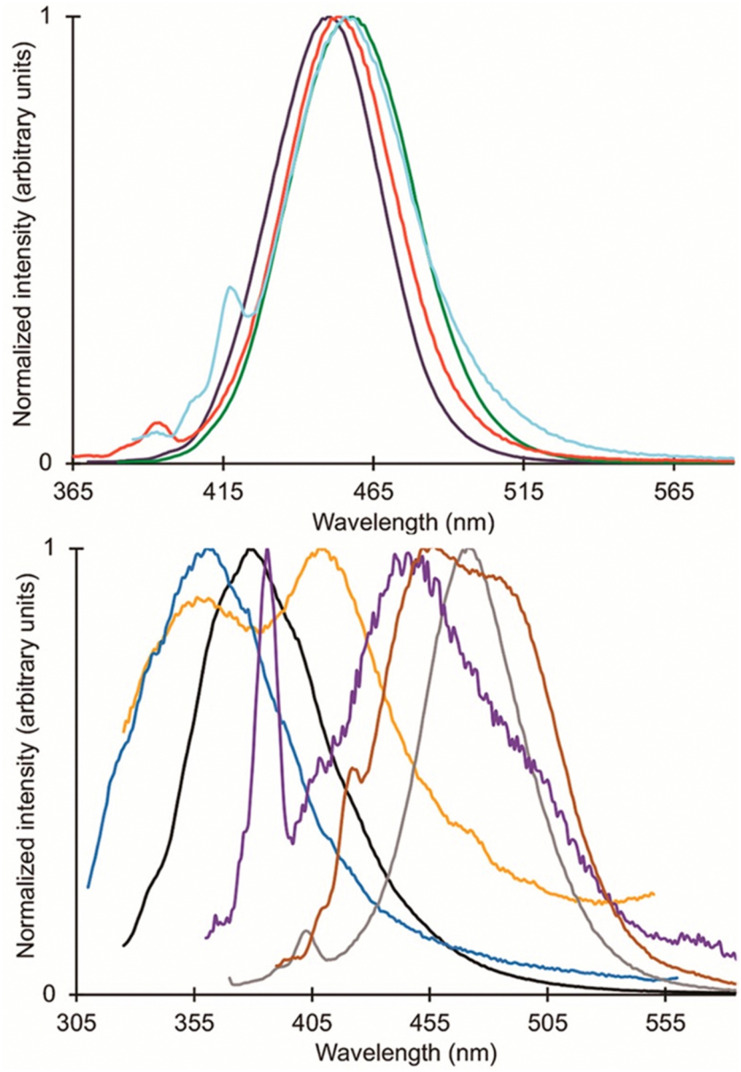
Emission spectra of Eu^II^1 (top) and Eu^II^28 (bottom). Color code for 1: red = [Eu(1)(NO_3_)_3_]I_0.83_[NO_3_]_0.17_; green = Eu(1)(OTf)_2_; dark-purple = [Eu(1)I]I; and cyan = Eu(1)(NCS)_2_. Color code for 28: blue = Eu28(NO_3_)_2_; black = Eu28(OTf)_2_; purple = [Eu28I(CH_3_OH)]I; brown = [Eu28Br(CH_3_OH)]Br; and grey = (CH_3_OH)28(Eu(μ-Cl)(ZnCl_3_)_3_). Reprinted with permission from S. S. Bokouende, D. N. Kulasekara, S. A. Worku, C. L. Ward, A. B. Kajjam, J. C. Lutter and M. J. Allen, *Inorg. Chem.*, 2024, **63**, 9434. Copyright 2023 American Chemical Society.

Following studies with 12 and 18, further studies to tune Eu^II^ electronic properties were undertaken using another ligand, tris[2-(2-methoxyethoxy)ethyl]amine, 28.^87^ Ligand 28 is structurally similar to 1, but lacks one tertiary amine, making it acyclic and more flexible than 1. Eu^II^28 was of interest to investigate how flexible acyclic ligands influence the properties of Eu^II^ with respect to coordination number, geometry, bond length, and luminescence spectra. Within this study, 1 and 28 were complexed with Eu^II^ and studied initially in the solid state. Complexes of Eu^II^1 contain Eu^II^ with coordination numbers of ten and square antiprism geometries, and complexes of Eu^II^28 contain Eu^II^ with coordination numbers of nine with muffin geometries.^75,87^ The solid-state structures show slight differences in bond lengths and bond angles of Eu^II^1 and Eu^II^28, which were likely due to differences in counterion and solvent molecule binding between each complex. To further understand coordination environment with different counterions, absorption and luminescence spectra of each complex in solution were studied (Fig. 9). The UV-visible spectra for Eu^II^1 and Eu^II^28 show almost no dependence on counterion identity for most complexes of Eu^II^1 and Eu^II^28, with absorbance peaks centered around 320 nm.^87^ For complexes of Eu^II^1, the emission and excitation spectra did not vary greatly between different coordinating counterions, indicating that different counterions do not influence the luminescence of Eu^II^1 significantly in acetonitrile. Despite the lack of shifts seen in excitation and emission spectra for Eu^II^1, complexes of Eu^II^28 with different counterions have a wide variety of emission and excitation wavelengths, spanning an emission maxima range of 355 to 525 nm. The wide range of wavelengths recorded implies that the flexibility of 28 enables innersphere interactions of counterions with Eu^II^, splits d-orbitals, and influences geometry in solution. This study highlights the difference between flexible, linear ligands, and macrocyclic ligands, and how that difference alters the properties of Eu^II^. The ligands studied for luminescence with Eu^II^ can also be used with other lanthanides to elicit similar trends in luminescence properties.^88,89^ Further, the luminescence studies of Eu^II^ have influenced ligand design for applications in catalysis, lighting, and imaging.

## Eu in catalysis

**Fig. 10:**
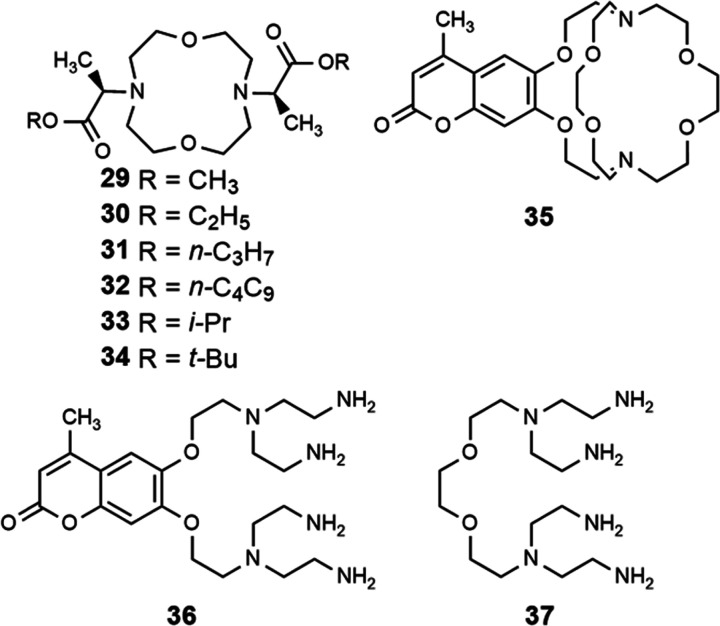
Ligands used for catalysis-based studies.

Catalytic studies in the Allen Research Group were initially inspired by luminescence-decay measurements of Eu^III^ toward understanding catalytic mechanisms. Additionally, luminescence studies of Eu^II^ led to studies of photoredox catalysis with the ion. Ligands described in this section relevant to catalysis influence are depicted in Fig. 10. Within this section, an overview of studies about the role of Eu^II^ and Eu^III^ in catalytic mechanisms and ligand design are described.

### Eu^III^ precatalyst studies

One of the first studies of catalysis in the Allen Research Group involved understanding the role of Eu^III^ in the Mukaiyama aldol reaction.^90^ The Mukaiyama aldol reaction uses Lewis acids, including Eu^III^(OTf)_3_ (OTf = trifluoromethanesulfonate), as catalysts to form carbon–carbon bonds in aqueous solution.^91,92^ Importantly, Eu(OTf)_3_ is able to catalyze this reaction in aqueous conditions.^93^ The mechanism of how OTf dissociates from Eu^III^ was studied by monitoring luminescence decay during the Mukaiyama aldol reaction to understand water coordination to Eu^III^. Luminescence decay is an analytical technique to measure water-coordination numbers of Eu^III^ and Tb^III^. The technique was pioneered by Horrocks and others,^94–97^ and it is commonly used in the study of contrast agents for MRI to measure water-coordination number. In applying the technique to aqueous catalysis, information about the behavior of Eu^III^(OTf)_3_ as a precatalyst in aqueous solutions can be obtained.^90^ In 100% water solutions, luminescence measurements showed that Eu^III^ coordinates to about eight water molecules. In 20% or less water in tetrahydrofuran solutions, roughly five water molecules were coordinated to Eu^III^, indicating that OTf almost completely dissociates. To understand how benzaldehyde, a reactant in the Mukaiyama aldol reaction, interacts with the Eu^III^ catalyst, benzaldehyde was spiked into solutions of Eu^III^(OTf)_3_ at various ratios of water to tetrahydrofuran. In solutions of less than 1% water in tetrahydrofuran, benzaldehyde partially displaced water, indicating that benzaldehyde interacts with Eu^III^ during the reaction. The maximum overall yield for the Mukaiyama aldol reaction using Eu^III^(OTf)_3_ was at 20% water in tetrahydrofuran, which is likely because there is the most interaction with benzaldehyde in that solvent due to few OTf ions coordinating to Eu^III^.^90,98^ Overall, this study demonstrated how luminescence measurements of Eu^III^ can be useful in elucidating catalytic mechanisms.

### Ligand design for Eu^III^-containing precatalysts

**Fig. 11:**
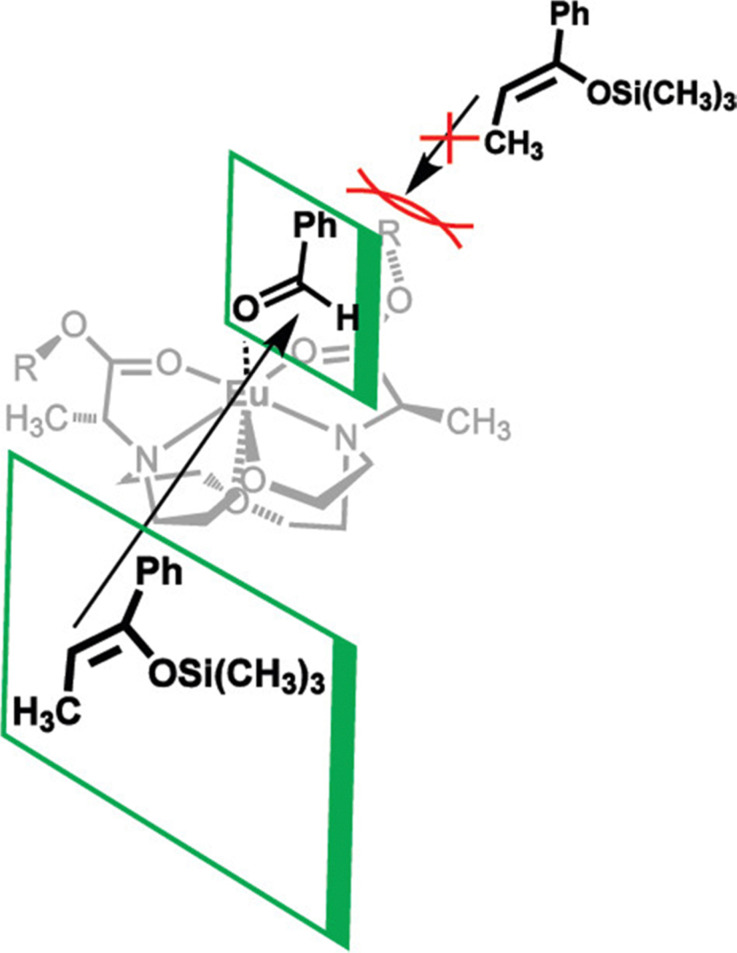
Proposed transition state for enantioselective catalyst and substrate binding for Mukaiyama–aldol reaction. Reprinted with permission from Y. Mei, P. Dissanayake and M. J. Allen, *J. Am. Chem. Soc.*, 2010, **132**, 12871. Copyright 2010 American Chemical Society.

In addition to adapting analytical techniques from MRI to study catalysis in aqueous solution, ligand design from MRI was adapted to synthesize enantioselective precatalysts.^99^ Ligands 29–34 were inspired by contrast agents involving 1,4,7,10-tetraazacyclododecane-1,4,7,10-tetraacetic acid. Modifications were incorporated to make the ligands chiral and provide open coordination sites to promote reactivity. Importantly, because of the labile nature of ligands with small denticities with trivalent lanthanides, large denticities are needed to maintain complexation in aqueous solution. The synthesized ligands had various ester groups attached with different chain lengths and bulkiness, that controlled substrate binding to Eu^III^ to improve enantioselectivity of reactions. The ligands synthesized had six sites for coordination to Eu^III^, multiple stereocenters, and *C*_2_ symmetry, to promote metal–substrate binding from either side of the precatalyst. Catalytic studies demonstrated that linear ester groups enable larger yields than bulky ester groups and improve enantioselectivity. To understand the catalytic mechanism, luminescence-decay measurements were acquired for all Eu^III^-containing complexes during catalytic reactions. When the benzaldehyde substrate was added into the reaction, water-coordination number to Eu^III^ decreased, implying benzaldehyde was coordinating to Eu^III^. Proposed transition states of benzaldehyde to Eu^III^-containing complexes suggests that the benzaldehyde substrate coordinates to Eu^III^ and is blocked by the ester group on one side enabling the other silyl enol substrate to only attack from one side, resulting in enantioselectivity (Fig. 11).^99^

To further understand the proposed transition state of Eu^III^-containing complexes and the aldehyde substrates, a variety of aldehyde and silyl enol ether substrates were used in different reactions to monitor enantioselectivity.^97^ The yields of the various substrates using Eu^III^-containing complexes as catalysts had some of the largest stereoselective yields reported in aqueous solution. Further support for the proposed transition state was obtained by demonstrating that as bulkiness of the aldehyde substituent increased, the yield decreased. One study focused on ligand design for Eu^III^-containing precatalysts, specifically how changing functional groups from esters to alcohols, carboxylic acids, and amides influences catalytic yields.^100^ From these studies, catalytic yields varied between 6 and 20% for ligands with alcohols, carboxylic acids, or amides due to the lack of steric bulk compared to previously studied ester groups. Overall, the results of these studies demonstrate how adapting ligand motifs from MRI enable the generation of highly enantioselective catalysts in aqueous conditions, where lanthanide complexes tend to dissociate, resulting in outstanding enantioselectivities of the aqueous Mukaiyama aldol reaction. These studies further demonstrate the utility of luminescence-decay measurements in elucidating catalytic mechanisms of Eu^III^-containing complexes.

### Photoredox catalysis

The previous section described the use of Eu^III^ as a Lewis acid catalyst. Observations of the luminescence of Eu^II^ inspired the use of that ion in photoredox catalysis. This section describes photoredox catalysis using Eu^II^, which uses light to excite Eu^II^ into a state where it becomes a potent reductant.

**Fig. 12:**
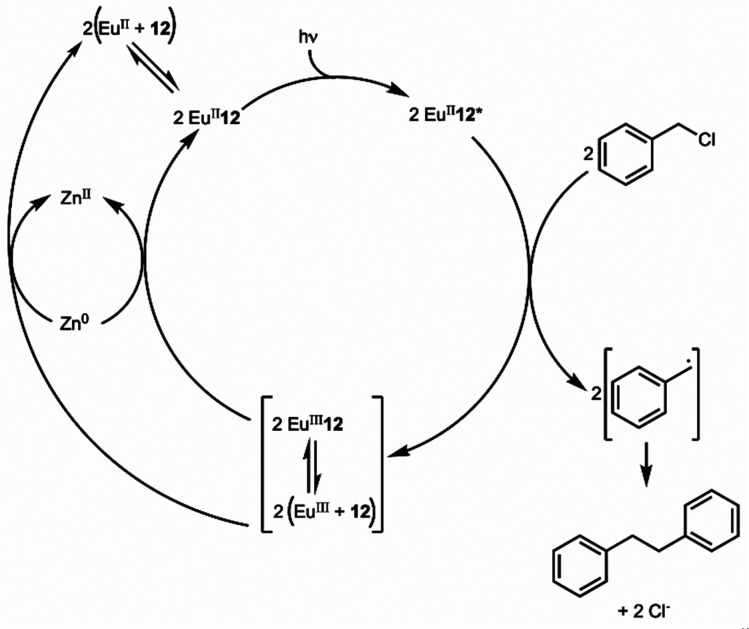
Proposed photoredox catalytic mechanism of Eu^II^12 for reductive coupling reactions of benzyl chloride.

Photoredox catalysis uses a redox-active catalyst to transfer an electron to a substrate to form products during organic reactions, typically using transition metals as catalysts.^101,102^ Previous luminescence studies of Eu^II^12 showed that the excitation and emission maxima were blue shifted compared to Eu^II^1, with luminescence lifetimes of 0.98 μs.^75^ Both of these qualities are desirable in photoredox catalysts, which led to studies using Eu^II^12 as a potential photoredox catalyst. First, the excited-state potential was calculated using cyclic voltammetry. The excited state potential of Eu^II^12 is −3.0 V *vs.* Ag/AgCl,^82^ which is more reducing than SmI_2_, a commonly used metal for photoredox catalysis.^103–105^ The reactivity of Eu^II^12 was tested using reductive coupling reactions of benzyl chloride to form 1,2-diphenylethane.^83^ An *in situ* mixture of one equivalent of Eu^II^, 12, and benzyl chloride were reacted in the presence of 460 nm blue light in methanol, which gave a 85% yield of 1,2-diphenylethane and 4.7% of toluene in 30 minutes. Control reactions revealed that no product formed when blue light, Eu^II^, or 12, were omitted, indicating all three components are needed to form product. To investigate why there were differences in quenching and product yields for the substrates, cyclic voltammograms were acquired of each substrate to find the cathodic peak to compare to the excited-state potential. The cathodic peak, indicating reduction, of (CH_3_)_3_CCl, C_6_H_5_Cl, CH_2_CHCH_2_Cl, and C_6_H_5_CH_2_Cl were −3.05, −2.93, −2.35, and −2.34 V, respectively *vs.* silver/silver chloride. Once the electron transfer mechanism was studied, catalytic reactions of Eu and 12 were investigated using benzyl chloride reductive coupling reactions. Within these reactions, EuCl_3_, 12, and Zn^0^ were used as catalytic precursors, with Zn as a sacrificial reducing agent. UV-visible absorbance and fluorescence measurements of EuCl_3_, 12, and Zn^0^ show that Zn^0^ reduces Eu^III^ to Eu^II^ but is not able to perform reductive coupling. Benzyl chloride reactions at 10 mol % of EuCl_3_ and 12 in blue light over six hours gave 80% yield of 1,2-diphenylethane and 11% of toluene. Catalyst loading was also studied by varying the amount of Eu^III^ and 12. At 5, 1, and 0.5% loading of Eu^III^ and 12, the yield of 1,2-diphenylethane was 71, 70, and 60%, respectively, with increasing catalytic turnovers at lower catalyst loading. As the catalytic loading was decreased, toluene yield increased. All reactions in this study were performed under anhydrous conditions, so to understand how small concentrations of water affected these reactions, EuCl_3_·6H_2_O was used in a wet glovebox, allowing water but no molecular oxygen within the atmosphere. The yield for the wet reaction was 80%, indicating that water does not affect the precatalyst. The proposed catalytic mechanism for this reaction is Eu^II^12 is excited by blue light to form Eu^II^12*. Eu^II^12* transfers an electron to benzyl chloride that ultimately leads to a reductively coupled product and oxidation of Eu^II^ to Eu^III^ and 12. Zn^0^ reduces Eu^III^ to restart the cycle (Fig. 12).^77^ This study showed the first visible-light promoted Eu^II^-based photoredox catalysis and was an important step in using Eu-containing complex for photoredox catalysis.

Beyond ligand 12, previously studied complexes of Eu^II^ that shifted the absorbance spectrum of Eu^II^ into the visible light region from the UV based on various ligand structures were studied to understand their ability as photoredox catalysts.^85^ To understand the excited-state potentials, cyclic voltammograms of Eu^II^19, Eu^II^21, Eu^II^22, and Eu^II^23 were obtained. The excited-state potentials of the complexes were between −2.3 and −3.2 V *versus* natural hydrogen electrode. More negative excited-state potentials are useful because they enable the reduction of a wide range of substrates. To study the catalysis-promoting ability of each complex, benzyl chloride coupling reactions were performed in blue light with 10 : 1 ligand-to-metal ratios to enable complex formation in solution. At large metal-to-ligand ratios, Eu^II^22 produced low yields and precipitated. The primary amine complex Eu^II^21 had the largest yields compared to secondary and tertiary amine-containing ligands. For Eu^II^21, moderate and large ligand-to-metal ratios produced good yields, but large ligand-to-metal ratios produced multiple alkylated byproducts. Comparisons of yields of Eu^II^12 and Eu^II^21 show that cryptates like Eu^II^12 are less likely to form Zn^II^-containing complexes due to the macrocyclic effect that improves overall product yield by increasing catalytic turnover. This study demonstrated the importance of ligand design for photoredox catalysts and how ligand structures and coordination chemistry can influence catalysis.

**Fig. 13:**
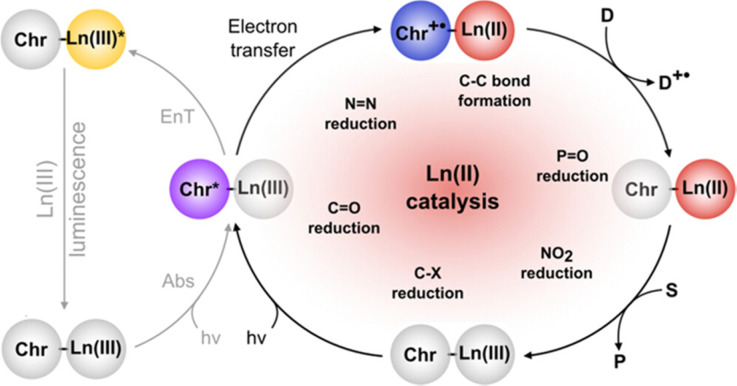
Proposed catalytic mechanism of chromophore-based complexes of Eu for organic reduction reactions. Reprinted with permission from M. Tomar, R. Bhimpuria, D. Kocsi, A. Thapper and K. E. Borbas, *J. Am. Chem. Soc.*, 2023, **145**, 22555. Copyright 2023 American Chemical Society. Licensed by Creative Commons CC-BY 4.0.

Eu^II^-based photoredox catalysis was reported by Borbas and coworkers using chromophore-based ligands.^106^ They investigated how chromophores excited by blue light transfer energy to reduce Eu^III^ to Eu^II^, to perform a variety of reductive couplings and functional group transformations. A series of three ligands with varying denticity and donor atoms were studied, with 35 and 36 having 6,7-oxycoumarin as a chromophore, and 37 having 7-aminocarbostyril as a chromophore. The excited-state potentials of these chromophores are more negative than −1.88 V *vs.* ferrocene/ferrocenium. These potentials are negative enough to reduce Eu^III^ to Eu^II^. To test the reactivity of Eu^III^35–Eu^III^37, several reductive coupling reactions with benzyl halide-based substrates and reduction reactions for sulfoxide, diazo, imine, azo, alkyl, phosphonate, aldehyde, ketone, and nitrile groups were performed. In most of these, Eu^III^35–Eu^III^37 was successfully reduced to Eu^II^35–Eu^II^37 resulting in excellent yields when in the presence of blue light and a sacrificial reductant (Zn^0^ or diisopropylethylamine). Control experiments showed that no product formed without Eu^III^, 35–37, or blue light. The proposed mechanism for this reaction is a photoinduced electron transfer from the chromophore to Eu^III^ results in the formation of Eu^II^ that transfers an electron to a substrate (Fig. 13). This mechanism was confirmed using electron paramagnetic spectroscopy. The results of these studies showed that Eu-based photoredox catalysts using a chromophore was capable of multiple reductive coupling and reduction reactions through photoinduced electron transfer.^106^

## Eu^II^ in separations

**Fig. 14:**
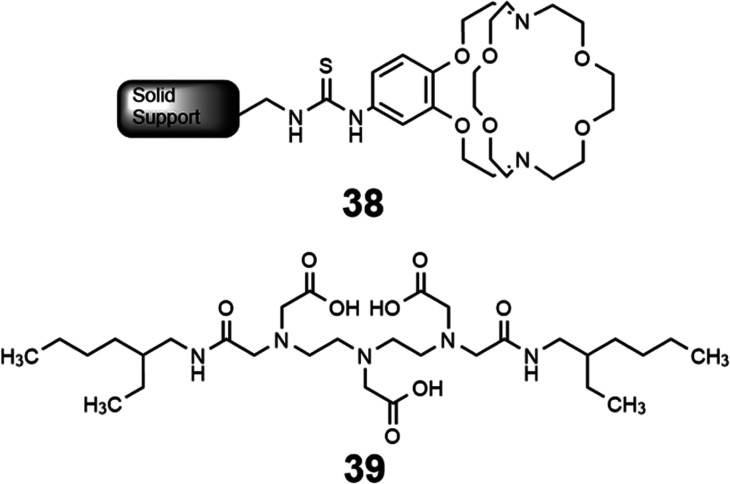
Ligands used for separations-based studies.

Applications of Eu, including MRI and catalysis, are only possible with stable feedstocks of the element. With the demand for rare-earth elements, including Eu, increasing because of their use in modern society, there is a need for efficient separation, extraction, and recovery of these elements. Common approaches for extraction involve sulfuric acid to leach lanthanides from ores or hydrochloric acid and heat to process rare-earth oxides to purify the elements.^107^ One limitation of these processes is that they generate considerable amounts of waste and are not highly selective for isolating target elements. Many developments in this area involve the principles of coordination chemistry to enhance the separation, extraction, and enrichment of rare-earth elements while attempting to minimize waste. An example by Binnemans and coworkers is the report of liquid–liquid extraction using nitrogen- or phosphorus-containing ionic liquids to trap Eu and then precipitate Eu salts with water.^108,109^ Aside from using ionic liquids, liquid–liquid extraction methods have also been studied with photochemical separation methods in alcoholic solutions to selectively precipitate lanthanides.^110^ Although the coordination chemistry of all the rare-earth elements is similar in many aspects across the series, methods have been developed to target the chemistry of individual elements.^111–113^ Some coordination chemistry factors that can be tuned to increase selectivity are the p*K*_a_ values of ligand donor atoms, kinetic inertness, thermodynamic stability, and ligand denticity. Ligands described in this section that influence coordination chemistry relevant to separations are depicted in Fig. 14.

Solid–liquid extraction is another method that uses coordination chemistry to separate rare-earth elements from each other.^114^ Solid–liquid extraction involves a solid support, which is often chemically modified with ligands, that collects metals from solution. After a metal ion of interest coordinates with the ligand on the support, and other metal ions are removed by washing, the metal ion of interest is removed from the ligand by washing with a different solvent. Solid–liquid extraction is often tuned through the ligand attached at the solid support, because ligands can bind rare-earth elements with a range of affinities. The interaction between the solid support and the ligand can also be tuned through covalent or noncovalent interactions. Solid–liquid extraction has gained interest because of its specificity and the small amount of waste it produces, and will be further discussed within this section.

**Fig. 15:**
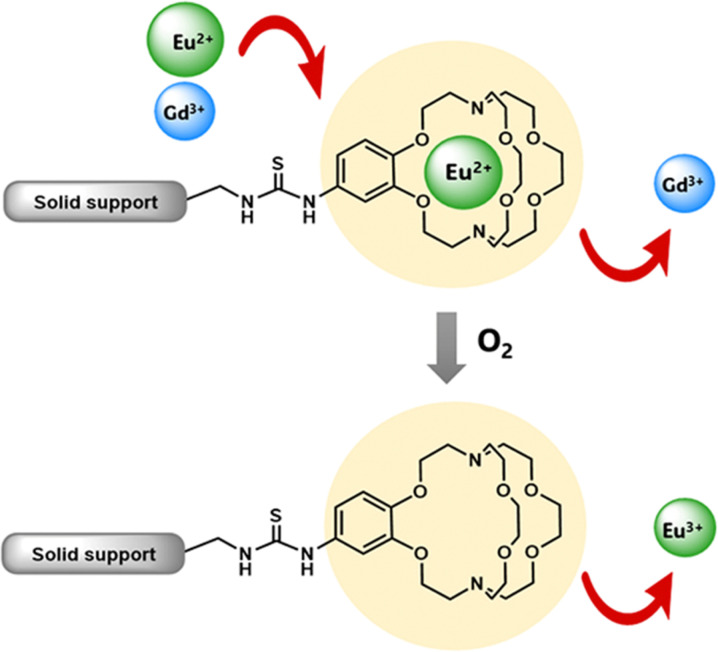
Benzo-2.2.2-cryptand covalently linked to a solid support, **38**, was used to selectively separate Eu from Gd. Reprinted with permission from D. N. Kulasekara, A. B. Kajjam, S. Praneeth, T. M. Dittrich and M. J. Allen, *ACS Appl. Mater. Interfaces*, 2023, **15**, 42037. Copyright 2023 American Chemical Society.

Some methods of separation specific to the isolation of Eu from other elements involve reducing Eu^III^ to Eu^II^,^115–122^ but those methods often require many steps to obtain high purity Eu. One collaboration with the Dittrich Research Group involved the hypothesis that 1 covalently bound to a solid support to form 38 would enable separation of Eu from Gd.^123^ This hypothesis is based on these two elements having similar coordination chemistries when they are both in the +3 oxidation state but having large differences in coordination chemistry when Eu is in the +2 oxidation state. The change in coordination chemistry between Eu^II^ and Gd^III^ arise from differences in atomic radii and charge density. Further inspiration for this hypothesis stems from reports that show 1 selectively binds divalent lanthanides over trivalent lanthanides.^36^ The atomic radius of Eu^II^ enables the ion to fit within 1 and coordinate more effectively with faster rates of association and slower rates of dissociation than Eu^III^.^36^ The resulting thermodynamic and kinetic differences between Eu^II^ and trivalent lanthanides with 1 increase the efficiency of separations.^123^ To separate Eu and Gd using solid-supported cryptands, a mixture of Eu^II^ and Gd^III^ was passed through solid NovaPEG resin that was covalently functionalized with 1 to form 38 (Fig. 15) which had a greater affinity for Eu^II^ than Gd^III^. After Gd^III^ was eluted, the Eu^II^-loaded support was exposed to air to oxidize Eu^II^ to Eu^III^, causing the atomic radius to change, releasing it from the solid support. The purity of the recovered Eu^II^ ranged from 86.7 to 99.3% after a single pass through the solid support. Solid support 38 is most effective between pH 3 and 5.5. Overall, the cryptand-modified solid support enabled isolation of high-purity Eu from a mixture of Eu and Gd.

**Fig. 16:**
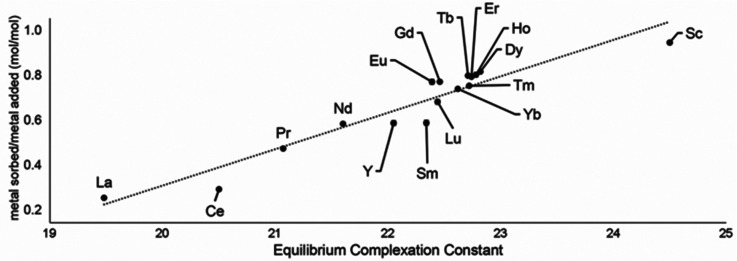
Metal sorbed onto 39 as plotted as a function of complexation constants of 13 for rare-earth elements at pH 3.3. Reprinted with permissions from Hovey *et al.*,^124^ copyright 2021, with permission from Elsevier.

Following studies using 38 for solid–liquid separations of Eu^II^, another study reported noncovalently solid-associated diesters of diethylaminetriaminepenta-acetic acid, 39, to separate rare-earth elements based on the affinity of 39 for the heavy rare-earth elements at an acidic pH.^124^ Ligand 39 was selected for this separation because it binds strongly to lanthanides and is reusable. Ligand 13 was functionalized with ethylhexylamine to form 39 that differs from 13 by the incorporation of two hydrophobic amides. To test the separation efficiency of 39, a solution containing 16 rare-earth elements (only Pm is excluded), 5 ppm each, was prepared. The results of the separation showed that 39 elutes the elements in the same order as the affinity 13 for the elements (Fig. 16). Solid support 39 is 130 times more effective at separating rare-earth elements at pH 3.3 than unmodified media.

**Fig. 17:**
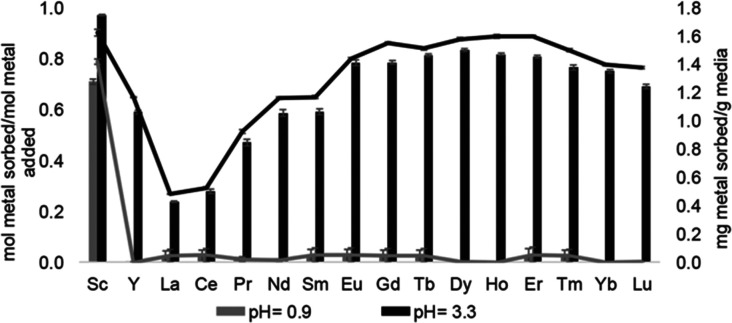
Sorption at pH 0.9 and 3.3 using 39. Reprinted with permissions from Hovey *et al.*,^124^ copyright 2021, with permission from Elsevier.

Solid support 39 shows a tendency to bind to heavy rare-earth elements during separations using coal-fly ash that contains many different elements. Unlike other systems, 39 has shown the ability to selectively bind heavy rare-earth elements over Ce and La (Fig. 17), two common elements that can make separations more challenging. This extraction of heavy rare-earth elements using 39 is achieved without the use of organic solvents during the separation, an issue with modern separation methods. This system has demonstrated effectiveness at recovering rare-earth elements from waste products with a preference for the rare-earth elements over Fe and Al, despite the concentration of metals. Further studies of this system showed improved enrichment of rare-earth elements in coal fly-ash from 0.024 to 10 wt%.^125^ The ligand involved in the system was also used to electrochemically remove Gd from samples with potential applications in hospital effluents.^126^

Another system designed to separate Eu from Yb uses inorganic tetrathiotungstate (WS_4_^−^) anions to induce electron transfers to the metal upon heating or exposure to light.^127^ Within this study, WS_4_^−^ reduces Eu^III^ to Eu^II^. When Eu^III^ is mixed with WS_4_^−^ in acetonitrile under ambient light, a Eu^II^-containing precipitate forms. The proposed mechanism of this precipitation is that Eu^III^ binds to WS_4_^−^, and once bound, light exposure induces an electron transfer from WS_4_^−^ to reduce Eu^III^ to Eu^II^, with the resulting Eu^II^-containing complex precipitating. The WS_4_^−^ separation technique was used on a complex phosphor mixture, resulting in 98.9% separation efficiency of Eu from the mixture. This study is an important step toward separation of rare-earth metals, showing the ability to separate metals from complex lanthanide mixtures like phosphors.^127^

Overall, solid support systems designed using the principles of coordination chemistry can selectively separate europium from other lanthanides using differences in oxidation state and pH, and these techniques can be extended to other lanthanides.^125,128^

## Conclusions

Throughout this article, information is presented regarding the differences in the coordination chemistry, magnetic properties, and optical properties of complexes of divalent and trivalent europium. Additionally, the influence of ligand design is described with respect to the tunable properties the of Eu ions and the relevance of these properties toward applications in MRI, luminescence, catalysis, and separation science. From these descriptions, several opportunities for future study arise. For example, translation of Eu^II^-containing contrast agents that exhibit kinetically controlled oxidation to biological systems that require systemic delivery is an area ripe for advancement. Also, with the prospect of increasing demand for rare-earth elements, more studies into separations of these elements from each other and from other elements will be needed. Further, gaps in knowledge of lanthanides largely fall in oxidation states other than +3, and to address those gaps, the study of other lanthanides in addition to Eu will be needed. Overall, continuing research using divalent and trivalent Eu, and the rest of the lanthanides, will aid in better understanding of the wide range of these ions in applications such as MRI, luminescence, catalysis, and separation science.

## Author contributions

All authors contributed to the writing, reviewing, and editing of this manuscript.

## Data availability

No primary research results, software, or code have been included, and no new data were generated or analyzed as part of this review.

## Conflicts of interest

There are no conflicts to declare.
